# Preparation of Imidazole Compounds as Latent Curing Agents and Their Application in RGB LED Packaging

**DOI:** 10.3390/ma17194935

**Published:** 2024-10-09

**Authors:** Jiangcong Chen, Shujuan Zhang, Biwen Li, Pinghu Chen, Hengfeng Li

**Affiliations:** 1 School of Materials Science and Engineering, Central South University, Changsha 410083, China; zhongnancong@163.com; 2 GYGAIR Intelligent Technology Co., Ltd., Guangzhou 510000, China; 3 Ji’an MuLinSen Industrial Co., Ltd., Ji’an 360800, China; shujuan.zhang@zsmls.com; 4 College of Mechanical Engineering, University of South China, Hengyang 421000, China; libiwen68@163.com (B.L.); chenpinghu1986@163.com (P.C.)

**Keywords:** RGB LED, non-conductive adhesives, chip–substrate joint, shear strength, light attenuation, reliability analysis, caterpillars

## Abstract

LED packaging miniaturization has raised more requirements for LED materials. As a material contacting the LED chip directly, the reliability of LED non-conductive adhesive has also garnered increasing attention. This study optimized the formula for non-conductive adhesives for an imidazole curing system. The optimized composition of the adhesive is 25%wt for the curing agent and 30%wt for the silica. The prepared non-conductive adhesive has a 7-day pot life and 9-month storage stability. The shear strength reached 87 g and 72 g at 25 °C and 160 °C, respectively. The reliability of the LED modules packaged with the non-conductive adhesive was researched. The green and blue light intensity change was 4.7%, 43.7%, respectively, indicating good anti-aging properties. The blue light decay was mainly due to adhesive aging. The non-conductive adhesive effectively prevented “caterpillar” growth. This provides useful and practical guidelines for industry for applications of adhesive in different packages.

## 1. Introduction

LED display technology is considered one of the most promising next-generation technologies [1]. Compared to conventional display technology, it offers advantages including power saving, high brightness, and high color saturation. Primarily used for indoor and outdoor large screens [2,3], LED displays are seeing growing demand for improved image quality and cost performance. Consequently, the pixel density, refresh rate, and scanning mode are constantly advancing. RGB LEDs serve as pixels in displays and are decreasing in size. The “caterpillar” phenomenon caused by metal migration within LEDs emerges under high refresh rates and scanning modes, which has become a bottleneck impeding high-quality LED display advancement.

The chip–substrate joint plays an important role in LED performance due to the direct contact with the LED chip [4]. Miniaturized LED chips require higher thermal conductivity, mechanical properties, and operability from chip–substrate joints than ordinary adhesives. Therefore, developing a high-performance LED chip–substrate joint remains an industry concern [5,6,7]. The ideal chip–substrate connection material should have a long shelf life, easy application, and adequate bond strength, and it should not chemically react with flux or solvent [8,9]. Among bonding methods, epoxy adhesives offer a higher shear strength, are useable in smaller spaces, have no short circuit issues, are useful in low thickness (<40 μm), are cost-effective, easy to apply, and easy to cure [10,11]. Thus, epoxy resins are widely used as chip–substrate adhesives and for low-to-medium performance LED packaging. For an epoxy resin system, curing agents significantly influence the cured epoxy properties. Generally, epoxy monomers can be cured with anhydrides, amines, imidazole, polyamides, onium salts, and carboxylic acid [12]. Among these, imidazole curing agents have drawn attention because epoxy resins cured with imidazole agents offer a high curing efficiency, a wide curing temperature range, and excellent mechanical properties [13,14,15].

In this research, imidazole derivatives were used to prepare a non-conductive adhesive. The curing reaction of epoxy resin and imidazole derivatives was conducted at various concentrations and investigated. The shear strength and expansion coefficient were studied. The reliability of the LED was studied from the aspects of photoelectric performance, moisture, heat resistance, and the corresponding interface microstructure, and the mechanism to prevent “caterpillars” was discussed.

## 2. Materials and Methods

### 2.1. Material

**Scheme 1:**
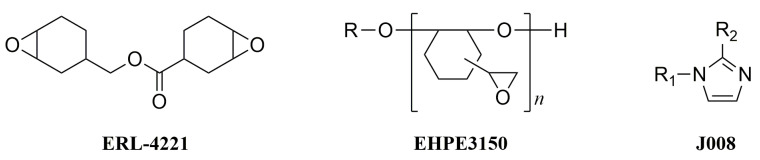
The chemical structure of the epoxy resins and the curing agent.

Cycloaliphatic epoxy resins (ERL-4221 and EP3150) were purchased from Daicel Co., Ltd., (Tokyo, Japan). The imidazole curing agent (J008) and silver-plated bracket were supplied by Ji’an MuLinSen Industrial Co., Ltd., (Ji’an, China). The chemical structures of all of these materials are illustrated in Scheme 1. Spherical silica with an average diameter of 2 μm was purchased from JiangSu Finetal Powder Technology Co., Ltd., (Yancheng, China). Alumina chips (0.10 mm × 0.18 mm) were supplied by Xiamen Changelight Co., Ltd., (Xiamen, China) All the reagents were used as received.

### 2.2. Preparation of Non-Conductive Adhesive

Cycloaliphatic epoxy resins (EP) (ERL-4221 and EP3150) were heated and stirred at 60 °C at a mass ratio of 7:3. Different concentrations of J008 and spherical silica were added sequentially to the EP mixtures at room temperature, preparing the adhesives for thermal and mechanical properties testing. The EP/imidazole mixtures were stirred for 10 min at 1500 rpm revolution and 900 rpm rotation using a double planetary centrifugal debubbling stirrer for DSC test. Shear strength test samples were prepared by adhering chips to silver-plated brackets using the adhesive. The curing process was 170 °C for 2 h.

### 2.3. Preparation of the RGB LED Package

The LED package sample was prepared on an automatic production line according to the RGB LED packaging process. Non-conductive adhesive was used as the die attach material for the blue and green chips. Three display modules were fabricated using patch technology, and the number of LEDs in each module was 24 K. A control group was prepared using the same method with a commercial non-conductive adhesive (S209F, KANEKA, Osaka, Japan).

### 2.4. Characterizations

Thermal dynamic analysis of the curing behavior for a non-conductive adhesive was performed using a differential scanning calorimeter (DSC 25, TA Instruments, New Castle, DE, USA) from 50 to 250 °C under a nitrogen atmosphere. The shear strength test determined the interfacial adhesion strength between the chip and the substrate. The sample with non-conductive adhesive and a blue light chip (0.1 mm × 0.18 mm) was attached to the LED silver lead frame, then heated to 170 °C for 2h. With this sample preparation method, a multifunctional bond tester (DAGE 4000, Nordson DAGE, Aylesbury, UK) determined the results of the interfacial adhesion strength tests at 200 μm/s and a test height of 35 μm under 25 °C and 160 °C, respectively. The coefficient of thermal expansion (CTE) value was determined by a thermal mechanical analyzer (TMA402F1, Netzsch, Germany) according to the standard ASTM D3386 [16]. The cured sample was cut into a size of 5 × 5 × 16 mm^3^. To ensure good contact between the probe and the samples, a force of 0.02 N was used. The specimen was scanned from 30 °C to 250 °C at a 5 °C/min rate. The morphologies of the chip–substrate joint in the LED samples were observed by an environmental scanning electron microscope (SEM, JSM-IT100, JEOL, Tokyo, Japan), and the surface morphologies of the LED were observed by a three-dimensional microscope (microscopes, VHX-970F, Keyence, Osaka, Japan). An aging experiment was conducted with a constant current aging machine inside a room temperature aging chamber. The red, green, and blue chips were lit for more than 6000 h at 3 mA, 5 mA, and 5 mA, respectively, using an IS standard test machine (instrument system CAS 140 CT, Munich, Germany) to measure the light intensity during the test. A temperature–humidity cycle test was conducted under an applied electrical load. The display modules were turned on in a programmable constant temperature and humidity test chamber. The temperature and humidity cycle conditions were as follows: 4 h at 85%/93% RH and then −40 °C for 4 h; the cycle test time was not less than 96 h. A commercial RGB LED with S209F as the chip–substrate joint (non-conductive) was used for the comparison test.

## 3. Results and Discussion

### 3.1. Curing Behavior of the EP/Imidazole Mixture

**Figure 1:**
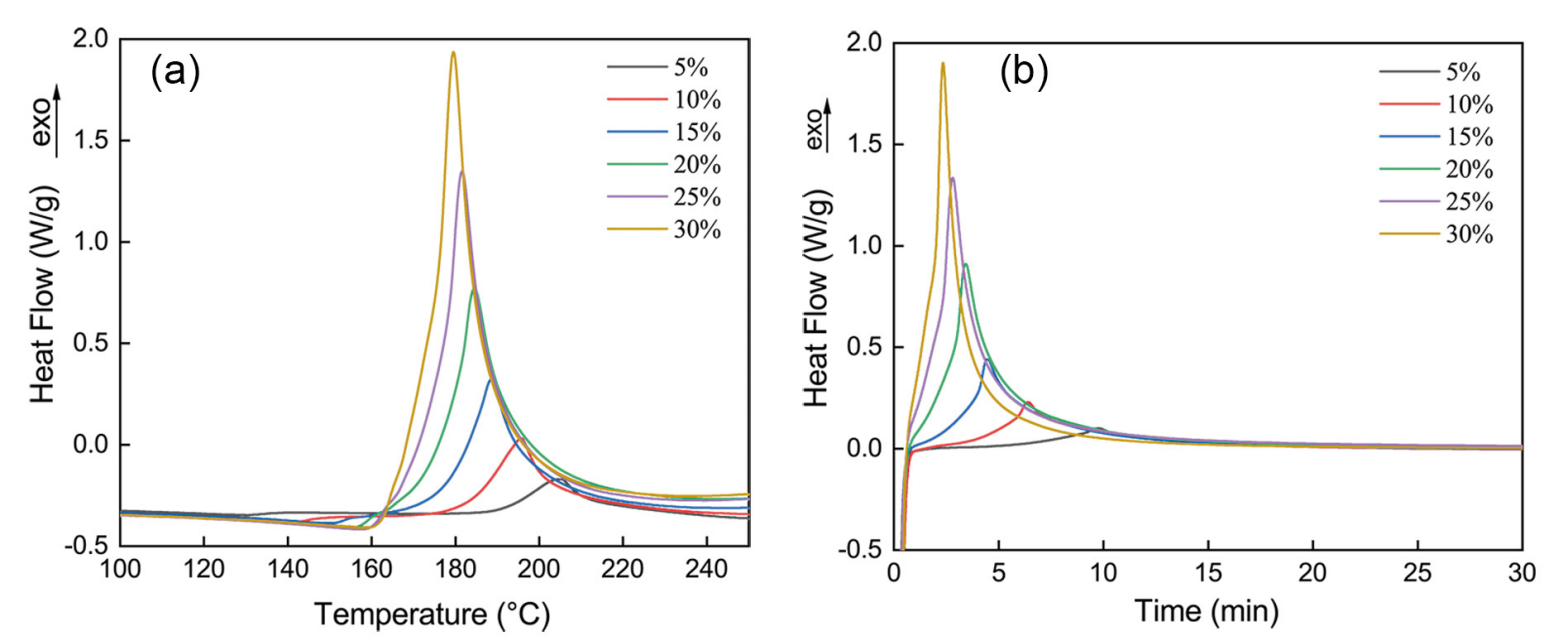
(**a**) Non−isothermal DSC curves of EP/J008 mixtures at different J008 contents; (**b**) iso−thermal DSC curves of EP/J008 mixtures at different J008 contents.

The curing behavior of EP systems containing 5~30 wt% imidazole derivative (J008) was investigated by differential scanning calorimetry (DSC). The non-isothermal and isothermal DSC curves of EP systems are shown in Figure 1. All the curves exhibited one exothermic peak, attributed to the anionic chain polymerization. This differs from previous research showing two exothermic peaks. Referring to the literature [17,18], the first exotherm is due to the addition reaction between the 1-position N-H of the imidazole and epoxy groups. The second exotherm results from the anionic chain polymerizations of epoxy groups initiated by the 3-position nitrogen of the imidazole ring. This difference may result from introducing a functional group to the 1-position of imidazole [19]. The curing exotherm of the EP/J008 mixtures noticeably shifted to a higher temperature relative to the conventional EP/imidazole. The electron-withdrawing and steric effects of the functional group reduced the nucleophilicity of imidazole’s 3-position nitrogen, restraining the reactivity of imidazole at a lower temperature. This caused the system to regain a higher curing activity with good thermal latency. Therefore, J008 is more suitable for one-component epoxy fabrication. The curing exotherm became more obvious with the increasing J008 content. A broad exotherm appeared at concentrations below 10%wt, while a sharper exotherm was observed above 25%wt. The peak maximum temperature and ΔH_tot_ of the EP/J008 systems shifted to a lower temperature with the increasing J1008 content. A higher curing agent ratio led to a faster and more complete reaction due to the greater heat release. The increasing ΔHtot in the non-isothermal DSC indicated the polymerization was a slow reaction, not completed during dynamic heating at 10 °C/min.

**Figure 2:**
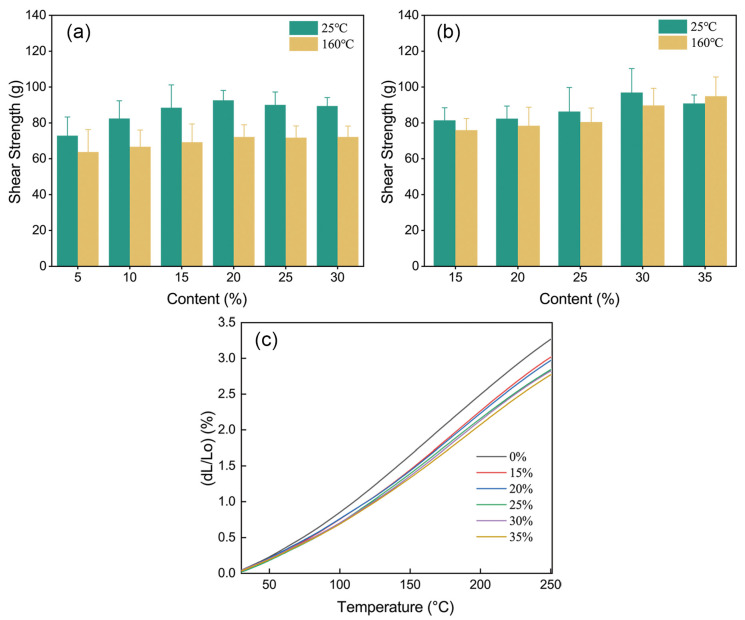
(**a**) Shear strength of the EP/J008 curing system with different J008 contents at 25 °C and 160 °C, respectively; (**b**) shear strength of the EP/J008-25% curing system with different silica contents at 25 °C and 160 °C, respectively; (**c**) the CTE of the EP/J008-25% curing system with different silica contents.

To further investigate the curing behavior of the EP/J008 system at different concentrations, isothermal DSC thermograms at 170 °C were obtained, as shown in Figure 2b. The exothermic heat (ΔH) increased with the increasing concentration and then stabilized. The DSC data for a heating rate of 10 °C/min and 170 °C isothermal are summarized in Table 1, including the starting temperature (To), peak temperature (Tp), and exothermic enthalpy of the curing reaction (ΔH). The ΔH approximately reflects the curing degree of the system, with a larger ΔH indicating a more complete reaction of the EP. The ΔH of the 25%wt and 30%wt J008 systems were similar, suggesting that the epoxy could fully cure at 25%wt J008 at 170 °C for 1 h. The gelation time of the EP/J008-25%wt was 6.33 min, while the EP/J008-30% was 3.47 min. The gel time of the EP/J008-30% was half that of the EP/J008-25%, with the initial reaction being too fast to form a cavity, affecting the bonding ability.

### 3.2. Shear Strength of the Adhesive

Shear strength is an important performance metric for LED chip-to-substrate joints. Given that the LED welding process requires heating to 160 °C, high-temperature shear strength is also important. The shear strength of the curing systems with different concentrations of J008 was investigated at 25 °C and 160 °C, as shown in Figure 2a. The results indicated the chip–substrate joint at 25 °C was generally greater than that at 160 °C. This is because 160 °C is close to the adhesive’s glass transition temperature, causing a softening and decline in the mechanical properties. The shear strength increased with the increasing J008 concentration at the same temperature, stabilizing around 25%wt. The shear strength at 25 °C enhanced from 72 g to 90 g. This was consistent with the thermal analysis. When the curing agent concentration exceeded 25%, the rapid reaction caused the internal stress to be unable to be released in time, resulting in the internal cracking of the chip–substrate joint and impacting the shear strength. Hence, 25%wt was the optimal concentration.

The addition of a filler to the adhesives provided an increase in the viscosity, storage, and loss moduli and imparted thixotropy to the adhesive. Spherical silica was chosen as the filler for its thermal stability. Figure 2b shows the shear strength of the EP/J008-25% at 25 °C and 160 °C with filler contents ranging from 15 to 35%wt. The shear strength of the samples with filler improved to some extent versus the samples without. With the increase in the silica content, the shear strength of 30%wt reached the maximum value and then showed a downward trend. This is because small amounts of active spherical silica particles filled the epoxy resin, evenly dispersing the internal molecular interaction and strengthening the bonding [20]. However, when the amount of the filler continued to increase, the proportion of epoxy resin in the whole adhesive liquid decreased, and the filler particles appeared to be “agglomerated”, resulting in a decrease in the adhesive liquid and bonding area. In this study, 30%wt of silica content yielded the highest adhesive shear strength, over 160 g and 89 g on average at 25 °C and 160 °C, respectively, meeting the 30 g shear strength required by the LED industry.

As seen in Figure 2c, with the increase in the silica content, the CTE decreased in both high- and low-temperature regions, indicating the addition of the filler improved the dimensional stability and reduced the CTE of the adhesive. The reason is that the expansion coefficient of spherical silica powder is smaller than that of the resin; so, the overall shrinkage rate of the dense structure formed by mixing the small filler particles with the resin is reduced. Combined with the shear strength experiment, when the silica content was 30%wt, the CTE in the selected characteristic temperature regions was 106 ppm/°C in the low-temperature regions and 152 ppm/°C in the high-temperature regions. The shrinkage performance in the low-temperature regions was better than that of products on the market. Therefore, the CTE of the prepared chip–substrate joint meets the application requirements of the product.

### 3.3. Comprehensive Properties of the Non-Conductive Adhesive Using in LED

According to the optimal concentration of the curing agent and the filler determined by the above experiment, a non-conductive adhesive using an LED with moderate viscosity and wide process adaptability was prepared with the addition of various additives such as a coupling agent (2%wt), a dispersant (2%wt), and a thixotropic agent (6%wt), which was denoted as YF003. Its comprehensive properties are shown in Table 2.

### 3.4. Light Attenuation of the Aged RGB LED

**Figure 3:**
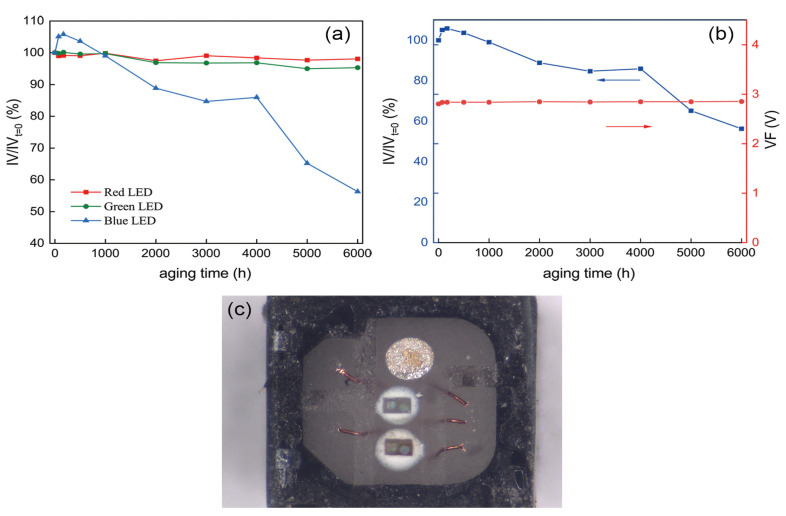
(**a**) The curves of luminous maintenance of RGB LED packages during aging; (**b**) the curves of luminous maintenance and forward voltage (VF) of blue LED lamps during aging; (**c**) metallographic diagram of YF003 after aging.

LED displays comprise many LEDs, meaning a few failures to emit light or a changing brightness will be noticed by users; so, the aging parameter is an important performance indicator of an LED. The photoelectric parameter changes in red, green, and blue LEDs during natural aging were investigated. The effect of the chip–substrate joint on the LED brightness during use is mainly reflected by yellowing and heat dissipation. As shown in Figure 3a, the luminous intensity of the blue LED was seriously attenuated (43.73%), while that of the green and red LEDs decreased only slightly after aging for 6000 h under the same conditions. The red chips were bonded with silver adhesive, which has excellent thermal conductivity. The slight attenuation (4.7%) of the green LED after natural aging showed that the non-conductive adhesive had good anti-aging properties. The blue LED luminous intensity initially exceeded the initial value, meaning a brighter light emission. This could be a defect annealing in the blue LED chips reducing the defect concentration and improving the luminous intensity of LED devices. The forward voltage (VF) barely fluctuated during lighting, while the luminous intensity of the blue LED lamps showed an obvious decrease (Figure 3b), indicating that the performance of the blue LED devices had little impact during light aging. The blue LED attenuation might be a short wavelength and high-energy blue light arousing degradation or other reactions in the chips or packaging materials, leading to serious light attenuation. Therefore, the significant light attenuation of the blue LED devices may be related to the degradation. After lighting the RGB LEDs at a current of 20 mA for more than 1000 h under room temperature, the luminous intensity of the blue LED was depreciated by almost 40%, while that of the red and green LEDs remained almost unchanged. FTIR spectroscopy showed that the aging of the packaging materials mainly occurred on the surface of the blue LED lamp [21]. To confirm this, the blue LED devices were cut open after 6000 h aging, as seen in Figure 3c; the chip–substrate joints and packaging adhesive under the green chips were normal-colored, while the packaging adhesive above the blue chips turned yellow, decreasing the blue light brightness.

### 3.5. The Reliability of Non-Conductive Adhesive under Rapid Temperature Change

**Figure 4:**
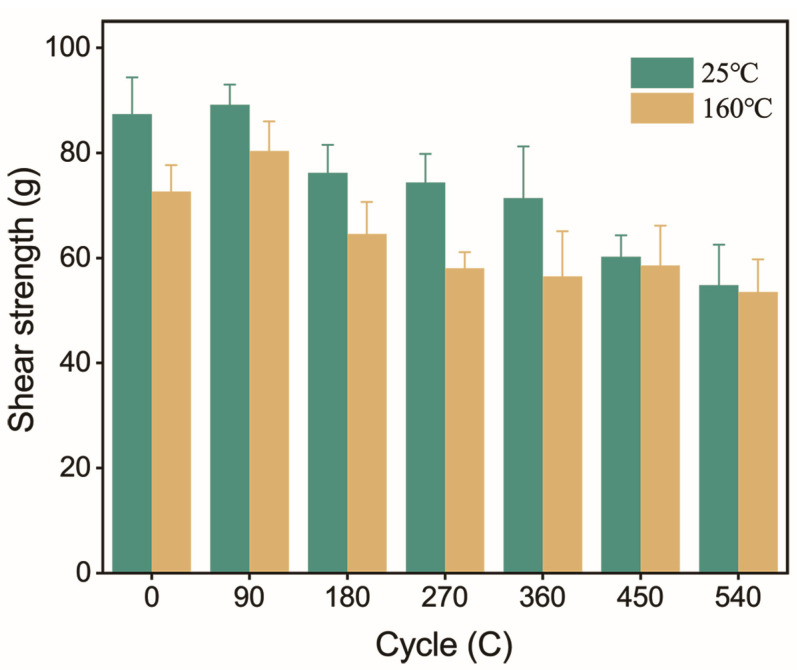
Shear strength after aging for different cycles under rapid temperature change.

A rapid temperature change test was used to confirm the reliability of the non-conductive adhesive. As shown in Figure 4, after 540 cycles of rapid temperature change, the shear strength of the chip–substrate joint showed a decreasing trend. The shear strength decreased by 37% from 87.34 g to 54.82 g at 25 °C and decreased by 26.5% from 72.66 g to 53.30 g at 160 °C. This is mainly due to the large difference in the coefficient of thermal expansion (CTE) between the non-conductive adhesive and the metal substrate; thermal stress will be generated during the heating process, resulting in the delamination between the materials.

### 3.6. “Caterpillar” Prevention

**Figure 5:**
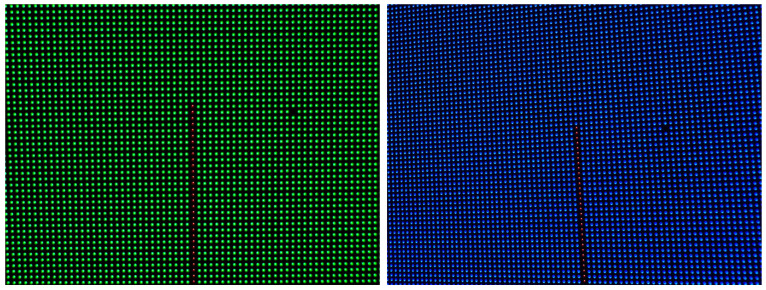
The caterpillar phenomenon of the display screen.

An LED is a humidity-sensitive device. Under a power supply, external moisture entering the LED will cause a “caterpillar” phenomenon. It has the following colors as shown in Figure 5.

**Figure 6:**
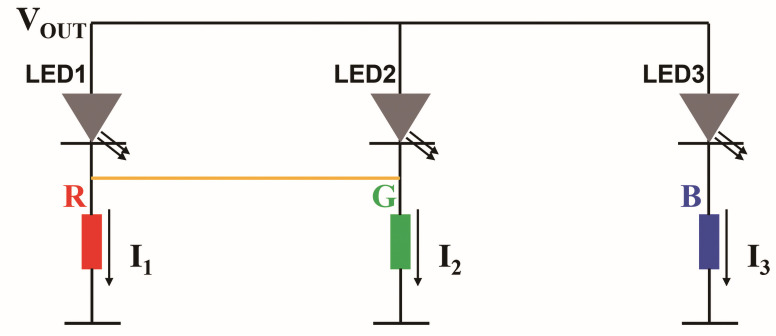
Schematic diagram of LED circuit.

The main reason for the “caterpillar” phenomenon is that the red chip and green chip or the red chip and blue chip are connected, and part of the voltage of the blue chip or green chip is allocated to the red chip with a lower forward voltage. Depending on the degree of conduction, the red chip and the blue chip or the green chip show different brightnesses. When the conductivity is low, the red chip is slightly brighter, and the blue or green chip is slightly darker. When the conductivity is high, the brightness of the red chip increases, and the blue or green chip gradually darkens until it is not bright at all. Its principle is shown in Figure 6.

**Figure 7:**
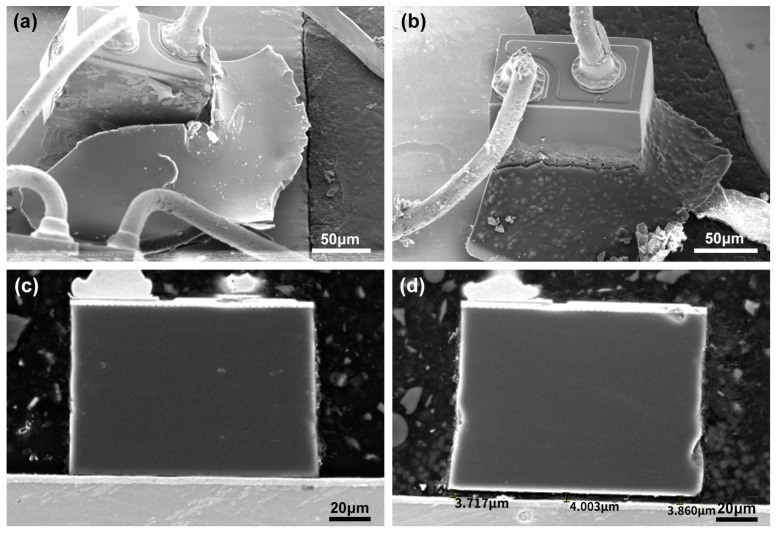
(**a**) Internal morphology of control group; (**b**) internal morphology of experiment group; (**c**) microsection of control group; (**d**) microsection of experiment group.

LED modules packaged with S209F (commercial non-conductive adhesive) and YF003 were selected for temperature- and humidity-dependent reliability analysis and a low-temperature conversion experiment. S209F is a filler-free cationic curing system. The LED packaged with S209F was named the control group, and the LED packaged with YF003 was named the experimental group. After the experiment, “caterpillars” appeared in the control group but not in the experimental group. We unsealed the two LEDs, as shown in Figure 7. The surface of S209 was smooth, and there were blocky foreign bodies on the side of the LED chip. The composition of Ag was tested via an energy spectrum. In the experimental group, the filled particles were clearly visible, and the sides of the chip were smooth. In this study, a silver-plated substrate was used, and the silver ion is an active metal; so, internal metal migration occurs in the presence of an electric field and water vapor. The directional movement of silver ions on the control group’s substrate to the side of the chip makes the common positive electrode of the LED and the negative electrode of the red chip conductive [22].

When the LED is powered on, the electrodes of the LED chip and the metal functional area of the substrate are equivalent to two electrodes of the electric field. An electric field is formed by adding a dielectric between the two electrodes. According to the equation [23], E = U/d, (where E is the electric field intensity, U is the potential difference between two points in a uniform electric field, and d is the distance between the two points), the smaller ε or d is, the larger E will be. The S209F does not contain fillers, and during the die bond process, the chip–substrate joint at the bottom of the chip was pressurized, resulting in direct contact between the chip and the silver-plated layer of the support, as shown in Figure 7c. Silica filler was added to YF003, completely wrapping the chip in the chip–substrate joint. The average distance between the bottom of the chip and the substrate increased to more than 2 μm, as shown in Figure 7d. Compared with S209F, the electric field intensity was smaller, which effectively delayed the metal migration on the side and reduced the “caterpillar”.

## 4. Conclusions

In this study, imidazole derivatives were utilized as curing agents to prepare a latent non-conductive adhesive for bonding blue and green chips in an RGB LED. The optimized composition of the adhesive was 25%wt of the curing agent and 30%wt of the silica. The prepared non-conductive adhesive had a 7-day pot life and 9-month storage stability. The shear strength reached 87 g and 72 g at 25 °C and 160 °C, respectively. The adhesive showed excellent anti-aging performance, with light attenuation of the green LED being only 4.7% after 6000 h of natural aging, and the shear strength decreased by merely 37% after 540 cycles of rapid temperature changes. Moreover, this non-conductive adhesive increased the distance between the electrodes of the LED chip and the metal functional area of the substrate, reducing their electric field strength and effectively preventing the “caterpillar” phenomenon in the display field.

## Figures and Tables

**Table 1:** Exothermic properties of the epoxy curing system at different J008 contents.

J008 Content (wt%)	Non-Isothermal DSC			Isothermal DSC	
	$T_{o}$ (°C)	$T_{p}$ (°C)	$∆H_{tot}$ (J/g)	$∆H_{tot}$	Gelation Time (min)
5%	190.3	205.2	23.7	58.03	/
10%	183.0	195.7	41.3	83.55	35.56
15%	178.9	188.5	64.6	123.60	18.01
20%	177.3	184.5	106.5	194.46	14.15
25%	175.4	181.6	144.0	232.01	6.33
30%	173.5	179.6	170.5	233.55	3.47

**Table 2:** Main properties of the non-conductive adhesive (YF003).

Item	Typical Value
Shelf life, at −20 °C	9 (month)
Pot life, at 25 °C	7 (day)
Viscosity, CAP2000+ Viscometer, at 25 °C	33,470 (mPa·s)
Thixotropic index	3.97
Shear strength	87 g (25 °C)
	72 g (160 °C)

## Data Availability

Data openly available in a public repository.

## References

[1] Lv X., Loo K.H., Lai Y.M., Tse C.K. (2014). Energy-saving driver design for full-color large-area LED display panel systems. IEEE Trans. Ind. Electron..

[2] Erdem T., Demir H.V. (2019). Color Science and Photometry for Lighting with LEDs and Semiconductor Nanocrystals.

[3] Bispo A.G., Saraiva L.F., Lima S.A.M., Pires A.M., Davolos M.R. (2021). Recent prospects on phosphor-converted LEDs for lighting, displays, phototherapy, and indoor farming. J. Lumin..

[4] Alim M.A., Aziz M.S.A., Abdullah M.Z., Kamarudin R., Lee J., Rusdi M.S., Khor C.Y. (2022). An experimental investigation of shear strength and adhesion on the chip-substrate joint in LED packaging. Int. J. Adhes. Adhes..

[5] Lee B.S., Yoon J.W. (2017). Die-attach for power devices using the Ag sintering process: Interfacial microstructure and mechanical strength. Met. Mater. Int..

[6] Lager M., Tews D., Haidar R., Hotz S., Cho W., Liong A., Brooks P. Advanced non-etching adhesion promoter for next generation IC packaging. Proceedings of the 2014 9th International Microsystems, Packaging, Assembly and Circuits Technology Conference (IMPACT).

[7] Manikam V.R., Cheong K.Y. (2011). Die Attach Materials for High Temperature Applications: A Review. IEEE Trans. Compon. Packag. Manuf. Technol..

[8] Alim M.A., Abdullah M.Z., Aziz M.S.A., Kamarudin R. (2021). Die attachment, wire bonding, and encapsulation process in LED packaging: A review. Sens. Actuators A Phys..

[9] Derebail A., Srihari K., Westby G. (1992). Knowledge-based Adhesive Selection for SMT Assemblies. Solder. Surf. Mt. Technol..

[10] Visakh P.M., Nazarenko O.B., Amelkovich Y.A., Melnikova T.V. (2016). Effect of zeolite and boric acid on epoxy-based composites. Polym. Adv. Technol..

[11] Sipaut C.S., Padavettan V., Rahman I.A., Mansa R.F., Dayou J., Jafarzadeh M. (2014). An optimized preparation of bismaleimide-diamine co-polymer matrices. Polym. Adv. Technol..

[12] Jin F.L., Li X., Park S.J. (2015). Synthesis and application of epoxy resins: A review. J. Ind. Eng. Chem..

[13] Lin K.-F., Wang W.-H. (1995). Imidazole and chromium acetylacetonate as additives for the cure and fracture toughness of epoxy resins and their composites. Polym. Compos..

[14] Ooi S.K., Cook W.D., Simon G.P., Such C.H. (2000). DSC studies of the curing mechanisms and kinetics of DGEBA using imidazole curing agents. Polymer.

[15] Mahendran A.R., Wuzella G., Kandelbauer A., Aust N. (2012). Thermal cure kinetics of epoxidized linseed oil with anhydride hardener. J. Therm. Anal. Calorim..

[16] (2000). Standard Test Method for Coefficient of Linear Thermal Expansion of Electrical Insulating Materials.

[17] Gajeles G., Lee S.H. (2019). Imidazole derivatives as thermal latent catalyst for thiol-michael reaction thermosetting resins. Eur. Polym. J..

[18] Cheng J.W., Wang J., Yang S., Zhang Q.Q., Huo S.Q., Zhang Q.X., Hu Y.F., Ding G.P. (2019). Benzimidazolyl-substituted cyclotriphosphazene derivative as latent flame-retardant curing agent for one-component epoxy resin system with excellent comprehensive performance. Compos. Part B Eng..

[19] Agren S., Mehdaoui R., El Haskouri J., Beyou E., Lahcini M., Baouab M.H.V. (2023). Reusable magnetic catalysed synthesis of fluorescent imidazole derivatives: Their use as chromogenic and fluorogenic probes for metal cation’s detection. J. Mol. Struct..

[20] Belli R., Kreppel S., Petschelt A., Hornberger H., Boccaccini A.R., Lohbauer U. (2014). Strengthening of dental adhesives via particle reinforcement. J. Mech. Behav. Biomed..

[21] Deng Z.T., Wang M.L., Zhu C.Z., Li C.H., Liu J.H., Tu M.L., Xie L., Gui D.Y. (2019). Study on light aging of anhydride-cured epoxy resin used for RGB LED packaging material. Polym. Test..

[22] Conseil-Gudla H., Jellesen M.S., Ambat R. (2017). Printed Circuit Board Surface Finish and Effects of Chloride Contamination, Electric Field, and Humidity on Corrosion Reliability. J. Electron. Mater..

[23] Liu X.L., Li C.Y., Yang T.H., Yin N.Q., Zhao G.L. (2024). Induced electric field intensity-enhancing water-drop triboelectric nanogenerator. Curr. Appl. Phys..

